# Evaluating the Outcome of an Unnecessary Request for CT Scan in Be'sat Hospital of Hamadan

**DOI:** 10.1155/2023/3709015

**Published:** 2023-02-22

**Authors:** Hossein Khosravi, Mohammad Hamidi, Safoora Nikzad, Leili Tapak

**Affiliations:** ^1^Department of Radiology, School of Allied Medical Sciences, Hamadan University of Medical Sciences, Hamadan, Iran; ^2^Department of Medical Physics, School of Medicine, Hamadan University of Medical Sciences, Hamadan, Iran; ^3^Department of Biostatistics, School of Public Health and Modeling of Noncommunicable Diseases Research Center, Hamadan University of Medical Sciences, Hamadan, Iran

## Abstract

**Aim:**

This study aimed to investigate the frequency of unnecessary tests requested in Be'sat Hospital in Hamadan.

**Materials and Methods:**

This descriptive research was conducted in order to investigate the frequency of unnecessary requests for CT scan and radiography of patients referring to the imaging department of Be'sat Hospital in Hamadan in a 4- to 6-month period. Patient information, including gender, age, type of CT scan test, the reason for requesting the test, the expertise of the requesting physician, and the result of the radiologist's report on each test, was extracted and collected.

**Results:**

A total of 1000 CT scans were evaluated. The mean age of these patients was about 36 years and most of them were men. The highest and lowest percentages of unnecessary cases were related to CT scans of the brain (42.3%) and facial bones (2.3%), respectively. The most and the least unnecessary CT scans based on the reason given for the request were related to multiple physical trauma (30.7%) and chronic kidney disease (1.5%), respectively.

**Conclusion:**

In all tests, over 74% of the reports were unnecessary and less than 26% were necessary. Therefore, it is necessary to reduce unnecessary requests to reduce the radiation dose of patients. Also, the knowledge of doctors should be increased in the field of appropriate evaluation of CT scan tests based on clinical guidelines.

## 1. Introduction

The diagnostic modalities used today, despite many scientific advances, are still not completely safe or entirely certain to be safe, such as MRI [1]. CT scan and radiology are widely used modalities in diagnostic processes. Due to the use of ionizing radiation, protection and safety points must be observed in using them.

Among the people who refer to the imaging departments, there are many people who do not seem to need this test at all, or perhaps with a better clinical examination; it might have revealed that there was no need to refer to the imaging departments and the request was completely unnecessary. The important question is, what are the factors leading to unnecessary requests? In the studies conducted, the most important factor and the main reason for the lack of clinical signs and symptoms to check the patient's condition is to see a doctor [2, 3].

According to Donelly's research in 2005, the most important reasons for unnecessary CT scan requests are doctors' obsession with stereotyping instead of clinical examinations, widespread psychological publicity for using the latest medical methods, and more financial gain from stereotyping. In addition to these reasons, many people tend to use imaging techniques that quickly announce results instead of multiple visits to physicians [4]. Based on Boodman's research in 2010, 30 to 50 percent of medical imaging is unnecessary and cannot provide useful information, and in order to reduce these requests, the correct use of diagnostic methods must be taught [5].

According to the FDA report, the most important factors influencing the preparation of unnecessary stereotypes are the lack of proper information about the radiation dose of the devices and the side effects of different imaging modalities and the lack of sufficient physician information about the patient, which may lead to repetitive or unnecessary stereotyping [6].

However, the most important and irreparable problem is the risk of unnecessary intake [7]. This problem is very evident in CT scan due to the use of higher radiation conditions, in a way that a scan of the pelvis and abdomen can give a person a basal dose of 3 years in a few moments, and the importance of the issue becomes apparent when a large part of this dose comes to sexual gonads. The risk of unnecessary radiation due to their hypersensitivity (infants are 10 times more sensitive to radiation) is much higher for children and infants [8].

The two principles of justification and optimization related to the general ALARA (As Low As Reasonably Achievable.) law must be observed in order to eliminate or reduce unnecessary radiation related to CT scan. This principle means that the relevant physician should consider the benefits and harms of performing these tests for the patient when requesting CT scans. By following this principle, the patient will receive less radiation dose [9–20].

In the field of evaluating the justification of imaging requests, especially CT scan, various studies have been conducted in different parts of the world [21–41], but in Iran, few studies have been conducted in this field. This study aimed at evaluating the justification for requesting various CT scan tests in Be'sat Hospital in Hamadan (one of the most important and busiest medical centers).

## 2. Methods and Materials

This study was performed to evaluate the frequency of unnecessary requests for CT scan and radiography of patients referred to the imaging ward of Be'sat Hospital in Hamadan. Patients' information including gender, age, type of CT scan test, the reason for requesting the test, a specialty of the requesting physician, and the result of the radiologist's report on each test was extracted and collected. The sample size included 50% of unnecessary medical imaging and a 95% confidence level, as well as 6% acceptable relative error, almost 1000 cases were obtained. Stratified random sampling (classes are formed according to the age or work experience of the specialist doctor) was obtained from among the patients who have performed various types of tests in a period of time. There were no restrictions on entering samples. In this study, the information of patients who have performed various CT tests was used, and no intervention was performed during the CT scan of the patient. Also, the process of collecting information did not cause any disturbance in the process of making appointments and the activity of the department.

Information on each test was collected based on the number of male and female patients who performed the test, the reason for the request, the average dose received, the financial cost, and the number of normal and abnormal results. Normal or abnormal results for each test were also recorded based on reports recorded by the radiologist. The number of positive reports (including abnormal findings in CT scans) for different tests is determined and divided by the total number of each test to determine the percentage of positive cases in different tests. Then, a relationship was determined between the percentage of abnormalities in different tests and the specialization of the requesting physicians so as to determine the situation of specialists in different fields in terms of observing the standard procedures of CT scan requests.

It should be noted that in this study, the information of patients who had previously performed various CT scan tests was used and no intervention was made in the process of performing CT scan of the patients.

The collected information was given to Excel software, and then, the analysis of the resulting data was carried out using SPSS software. In this regard, the mean and curvature of the standard, minimum, and maximum were used to describe quantitative indicators, and absolute and relative frequencies were used to describe qualitative variables.

## 3. Results

The CT scan tests of 1000 patients referred to the imaging department of Hamadan's Bes'at Hospital were evaluated in the 4- to 6-month period of the study. The average age of these patients is 35.94 years in the age range of (1–91 years), the majority of patients are male (64%), the type of test in 52% of patients is a CT scan of the brain, and the most requested CT scan is (38%) multiple physical injuries and 20% was the fall (Table 1).

**Figure 1:**
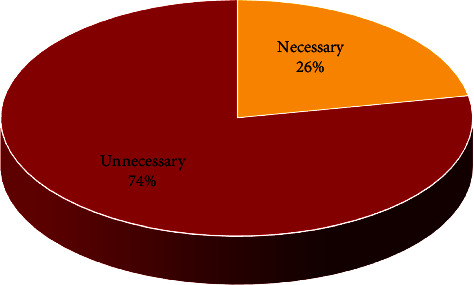
Displaying necessary and unnecessary findings in the CT scan images of various tests of the brain, sinuses, chest, abdomen, pelvis, and spine.

**Figure 2:**
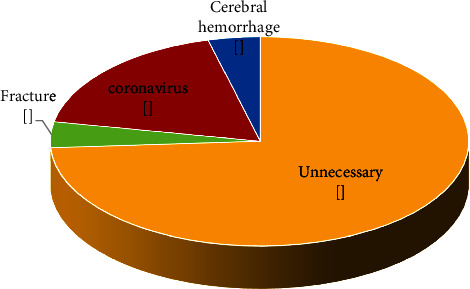
Displaying the percentage of essential and unnecessary items in CT scan images obtained from various tests of the brain, sinuses, chest, abdomen, pelvis, and spine.

The results of this study showed that 26% of the performed CT scans had abnormal findings (Figure 1). Also, the results reveal that 74% of the positive cases in the CT scan were unnecessary and 18% were corona, and fracture and brain hemorrhage both were reported at 4% (Figure 2). The effective doses are typical values for an average-sized adult. The actual dose can vary substantially, depending on a person's size as well as on differences in imaging practices. It should be noted that the average effective dose received by the patient per unnecessary CT scan was 4 mSv [16].

**Figure 3:**
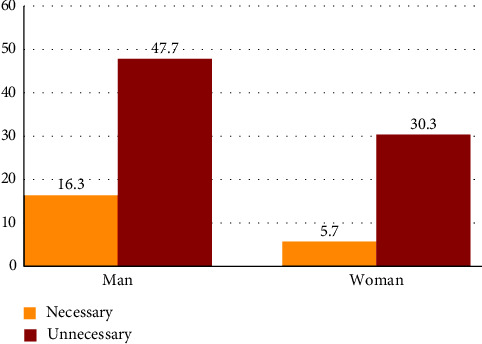
Correlation between unnecessary CT scan according to gender.

In the continuation of the investigations, the obtained results showed that performing unnecessary CT scans was significantly more in men than in women (*P* < 0.05). Because the number of men who had been referred to Be'sat Hospital due to trauma was higher than that of women (Figure 3).

**Figure 4:**
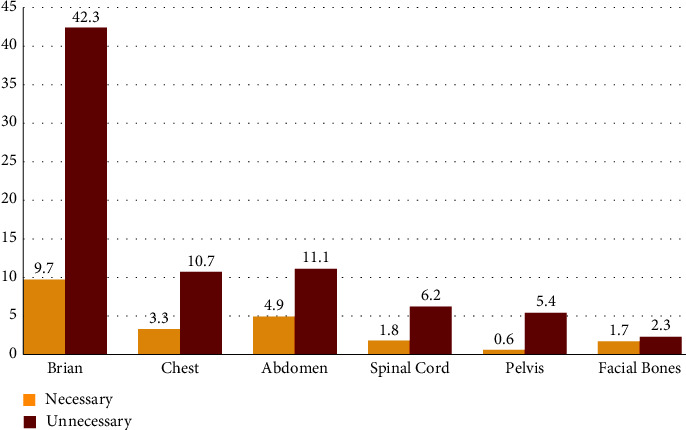
Correlation between unnecessary CT scans according to different CT scan tests.

Among the different CT scan tests performed on the patients, the results showed that the unnecessary CT scans of the brain were significantly more than other tests (*P* < 0.05) because the majority of patients referred to Be'sat Hospital suffered from trauma and nerve damage. (Figure 4).

**Figure 5:**
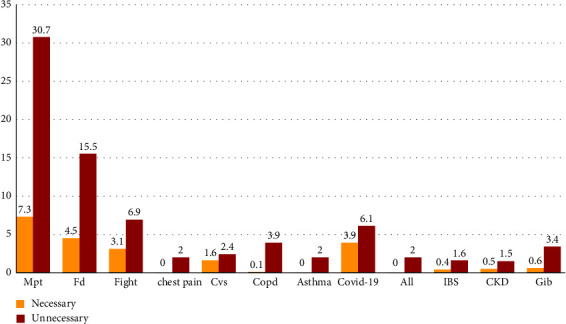
Correlation between unnecessary CT scans considering the reasons for the request.

In another study, the most unnecessary CT scans were evaluated to reveal the reasons for the request (Table 2). The results showed that the most unnecessary CT scans were related to Mpt and Fd with 73 and 45 cases, respectively. A statistically significant (*P* < 0.05) relationship was reported between the unnecessary CT scans and the reasons for the request (Figure 5).

## 4. Discussion

The present study showed that a large number of CT scan tests requested for patients were unnecessary. The highest and lowest CT scan results were related to CT scans of the brain and facial bones. In total, more than 74% of all CT scan tests performed on patients proved to be unnecessary. In other words, less than 26% of tests showed pathological cases. This is despite the fact that about 44% of the CT scan tests in Gunes Tatar et al. research were abnormal [29]. Of course, in the current study, the CT scan test for fracture was reported to be 4%, compared to the findings of Haghighi et al. [27], Bent et al. [28], and Wang and You [21], which were about 12%, 10%, and 14%, respectively, representing a more favorable situation.

According to AlQahtani and Kandeel research, in 2011 and 2012, there was an 18% increase in radiology requests compared to 2010. This standard for CT scan was reported at 23% at the same time. After examining 449 CT scan cases, this group concluded that about 30% of requests were unnecessary [11]. Hardman and Kahn stated that between 30 and 50 percent of medical imaging are unnecessary and cannot provide useful information, and to reduce these requests, the correct use of diagnostic methods should be taught [17]. In the study of Lehnert and Bree, out of 459 requested CT scans, 341 cases (74%) were necessary and 118 cases (26%) were unnecessary. At the end of the training of primary care doctors, he introduces the effective solutions in this field about the use of the Berd-Air image [30].

According to the report of the FDA organization, the most important factors affecting the preparation of unnecessary stereotypes are the lack of correct information about the radiation dose of the devices and the complications of different imaging modalities, and the lack of sufficient information from the doctor about the patient, probably causing the preparation of repeated or unnecessary stereotypes [18].

The results of this study showed that unnecessary brain CT scans and fractures were significantly more than other tests (*P* < 0.05). In the reference books, the initial abnormal CT scan in all patients with mild brain damage is mentioned about 20% [35, 36]. In the study conducted by the World Health Organization, on mild trauma, 5% of the CT scan is abnormal and if GCS is equal to 13, this percentage reaches 30% [37, 38]. In the study of Ibañez et al., 7.5% of abnormal CT scans were seen in patients with mild head trauma [39]. Therefore, in such patients, it is necessary to take more care to reduce the request for CT scan, and in hospitalized patients, since the sum of normal CT scans and cerebral edema is 88%, and that cerebral edema does not require surgery. Moreover, its treatment is medical. Therefore, for patients with mild brain damage who are not demented and whose general condition is improving and who do not have any fractures, there is more reason to consider CT scanning and it seems reasonable to undergo these patients. It is considered that if there are signs of neurological deterioration and deterioration of consciousness and deterioration of clinical symptoms, they should be examined by CT scan [40].

## 5. Conclusion

The general results of this study showed that most CT scans performed were normal (unnecessary). Since the limitation of cost control resources is one of the main considerations of the service delivery system, identifying the possible causes of unnecessary CT scans, including the lack of proper training of doctors regarding essential use criteria, the absence of national guidelines, and the absence of an audit and feedback system regarding the amount of use of radiology measures, it can provide doctors with a suitable platform for targeted interventions in line with the proper use of these measures.

## Figures and Tables

**Table 1:** Determining the number of CT scan requests for various tests of the brain, nasal sinuses, chest, abdomen, pelvis, and spine.

CT scan test type	Frequency	Percentage (%)
Brain	520	52
Chest	140	14
Abdomen	160	16
Spinal cord	80	8
Pelvis	60	6
Facial bones	40	4

**Table 2:** Determining the relationship between cases of unnecessary CT scans considering the reasons for the request.

The reasons for the request	Necessary cases	Unnecessary cases
Mpt^1^	73	307
Fd^2^	45	155
Fight	31	69
Chest pain	0	20
Cvs^3^	16	24
Copd^4^	1	39
Asthma	0	20
COVID-19	39	61
All^5^	0	20
IBS^6^	4	16
CKD^7^	5	15
Gib^8^	6	34

(1) Multiple physical trauma, (2) falling down, (3) cardiovascular strokes, (4) chronic obstructive pulmonary disease, (5) acute lymphocytic leukemia, (6) irritable bowel syndrome, (7) chronic kidney disease, and (8) castro-intestinal bleeding.

## Data Availability

The data used to support the findings of this study are available from the corresponding author upon request.
